# Testicular STAC3 regulates Leydig cell steroidogenesis through potentiating mitochondrial membrane potential and StAR processing

**DOI:** 10.1007/s00441-020-03312-8

**Published:** 2021-01-06

**Authors:** Xingyu Bi, Junfen Liu, Suming Xu, Yaoqin Wang, Xueqing Wu

**Affiliations:** Center of Reproductive Medicine, Children’s Hospital of Shanxi and Women Health Center of Shanxi, No. 13 Xinmin North Street, Taiyuan, 030013 Shanxi Province P.R. China

**Keywords:** SH3 and cysteine-rich protein 3 (STAC3), Steroidogenesis, Mitochondrial membrane potential, Testosterone, Steroidogenic acute regulatory protein (StAR)

## Abstract

SH3 and cysteine-rich protein 3 (STAC3), a small adapter protein originally identified as a core component of excitation–contraction coupling machinery, regulates the voltage-induced Ca^2+^ release in skeletal muscle. However, the possibility of additional, as yet unknown, non-muscle effects of STAC3 cannot be ruled out. Herein, we provide the evidence for the expression and functional involvement of STAC3 in spermatogenesis. STAC3 expression was localized in the testicular interstitium of rodent and human testes. By using the cytotoxic drug ethylene dimethane sulfonate (EDS), STAC3 expression was observed to be decreased sharply in rat testis after selective withdrawal of Leydig cells (LCs), and reappeared immediately after LCs repopulation, indicating that testicular expression of STAC3 mainly stems from LCs. From a functional standpoint, in vivo lentiviral vector–mediated suppression of STAC3 resulted in a significant decrease in testosterone production, and thereafter caused impairment of male fertility by inducing oligozoospermia and asthenospermia. The indispensible involvement of STAC3 in testicular steroidogenesis was validated using the in vivo knockdown model with isolated primary LCs as well as in vitro experiments with primary LCs. By generating the TM3^Stac3−/−^ cells, we further revealed that STAC3 depletion attenuated mitochondrial membrane potential and StAR processing in db-cAMP-stimulated LCs. Thus, the inhibitory effect of STAC3 deficiency on testicular steroidogenesis may be ascribed to a disturbed mitochondrial homeostasis. Collectively, the present results strongly suggest that STAC3 may function as a novel regulator linking mitochondrial homeostasis and testicular steroidogenesis in LCs. Our data underscore an unexpected reproductive facet of this muscle-derived factor.

**Electronic supplementary material:**

The online version of this article (10.1007/s00441-020-03312-8) contains supplementary material, which is available to authorized users.

## Introduction

Interstitial Leydig cells (LCs) are the main source for biosynthesized testosterone, and proper production of testosterone is essential for germ cell survival and differentiation (Goldberg, 1984; Holdcraft and Braun, 2004). Upon stimulation with luteinizing hormone (LH), cyclic adenosine monophosphate (cAMP) accumulates rapidly in LCs, where cAMP acts as an intracellular second messenger to regulate testosterone synthesis either acutely at the level of cholesterol transport or chronically at the level of gene transcription (Zirkin and Papadopoulos, 2018). In response to intracellular pulse by cAMP, steroidogenic acute regulatory protein (StAR) is expressed as a 37-kDa precursor outside mitochondrion, and is then imported into the mitochondrial matrix where it is processed into a mature 30-kDa form. StAR facilitates the transfer of cholesterol into mitochondrial matrix to fuel the testosterone biosynthesis. Therefore, StAR serves as the first and rate-limiting step of complicated testicular steroidogenesis (Stocco and Clark, 1996). Accumulated data suggest that the phosphorylation of StAR is critical in order to render the protein fully active in its capacity to support cholesterol transfer (Arakane, et al., 1997). Moreover, during aerobic respiration, ATP is generated and electrons are transported through enzyme complexes (nicotinamide adenine dinucleotide hydroxide dehydrogenase, succinate dehydrogenase, cytochrome bc_1_ and cytochrome oxidase) that are located at the inner mitochondrial membrane. This electron transportation yields free energy that generates a proton gradient. The proton gradient then promotes a pH differential (ΔpH) and a mitochondrial membrane potential (*ΔΨ*_*m*_), both of which provide the proton motive force driving ATP synthesis (Allen, et al., 2006). Collective findings suggest that proper ATP production generated by mitochondria is also crucial for steroid biosynthesis (Midzak, et al., 2011).

SH3 and cysteine-rich protein 3 (STAC3), a small adapter protein, was originally identified as a core component of excitation–contraction (EC) coupling machinery in skeletal muscle (Horstick, et al., 2013). It mediates voltage-induced Ca^2+^ release and contractility. Homozygous deletion of the *Stac3* gene results in complete paralysis and perinatal lethality with a range of musculoskeletal defects in mice (Nelson, et al., 2013). Likewise, mutations in the human *STAC3* gene lead to myopathy (Horstick, et al., 2013). To be noted, the biological effects of STAC3 known to date are largely performed in skeletal muscles. However, additional as yet unknown non-muscle actions of STAC3 could not be ruled out. In this regard, using panel-based nuclear and mitochondrial next-generation sequencing, aberrant expression of STAC3 has been associated with mitochondrial diseases in pediatric patients (Schoonen, et al., 2019). Novel expression of STAC has been reported in central nerve system (Polster, et al., 2018). Additionally, using *Xenopus* oocytes, Wu F et al. observe that Stac3 enhances expression of human CaV1.1, and helps to reveal gating pore currents in HypoPP mutant channels (Wu, et al., 2018). Nevertheless, the functional details of STAC3 in such non-muscle systems remain largely unexplored.

It is now evident that cellular Ca^2+^ homeostasis plays a central role in the modulation of diverse cellular functions in both germ cells and somatic cells within mammalian testis (Lee, et al., 2011). The identification of STAC3 as a potent regulator of Ca^2+^ homeostasis prompted us to evaluate whether this signaling is functionally expressed in testis. Our systematic analysis demonstrates that STAC3 may act as a novel factor linking mitochondrial homeostasis and testicular steroidogenesis, thus underscoring an unexpected reproductive facet of this novel adapter protein.

## Materials and methods

### Data mining

To explore the clinical relevance of *STAC3* expression, we performed data mining on the data sheet GSE45885, analysis on testicular biopsy specimens from normozoospermia (n = 4) or from non-obstructive azoospermia (n = 27) using Affymetrix Human Gene 1.0 ST Array (Malcher, et al., 2013). To computerize the expression level, raw files were imported into the GEO2R online software provided by the GEO database (Qian, et al., 2019).

### Human samples

Upon receipt of informed consent, normal human testicular specimens were collected from normozoospermic patients who underwent testicular biopsies during genital surgery procedures for varicocele or epididymal cysts (n = 12). Normal spermatogenesis was validated by histological examination and semen analysis (Tian, et al., 2014). Testicular tissues were immediately fixed in 4% paraformaldehyde (Sigma-Aldrich, Shanghai, China) and were embedded in paraffin. Partial samples were immediately snap-frozen in liquid nitrogen, stored at -80 °C for later biochemical analysis. The procedures involved in human study, strictly conformed to the 1964 *Helsinki Declaration*, were approved by the Human Research Committee of Children’s Hospital of Shanxi and Women Health Center of Shanxi (Approval #: CHSX-2014-00164b).

### Animal model

Experimental procedures for the humane treatment and well-being of the laboratory animals, in compliance with the Guidelines for the Care and Use of Experimental Animals from NIH, were approved by the Institutional Animal Care and Use Committee of Children’s Hospital of Shanxi and Women Health Center of Shanxi (Approval #: CHSX-2014-00164a). All surgeries in animals were performed under sodium pentobarbital anesthesia, and all efforts were made to minimize suffering.

C57BL/6 J male mice and 3-month-old male SD rats were obtained from the animal facility of our hospital. Animals were housed under environmentally controlled conditions (12 h light–dark cycle and 20–25 °C) with food and water provided ad libitum. They were allowed to acclimatize for at least 7 d before experiment.

Elimination of rat LCs was achieved by systemic administration of a single dose of ethylene dimethane sulfonate (EDS) (75 mg/kg i.p.) or vehicle control (dimethyl sulfoxide, water; 1.5:3.5, vol/vol), as described (He, et al., 2016). Testes and blood samples were harvested at 7, 14 and 28 d following EDS injection.

In vivo knockdown of Stac3 was carried out as described elsewhere (Kim, et al., 2010). After anaesthetization, testes were exposed and injected with 20 μl pLKO.1-CMV-tGFP-Stac3 (7 ng/μl) or empty vector (Sigma-Aldrich) using a fiber optics probe (diameter, 1.65 mm), under a Leica EZ4 dissecting microscope (Leica, Beijing, China). The testes were then returned to scrotum, incision was sutured, and mice were allowed to recover. Two days after microinjection, some mice were i.p. injected with testosterone propionate (TP, 3 mg/kg, Sigma-Aldrich) on a daily basis for consecutive 68 d. Testes and blood samples were harvested at 70 d after the first microinjection.

### Cell treatment

Primary LCs were isolated and purified according to a reported protocol (Riccetti, et al., 2017). Briefly, testes from adult mice were decapsulated, mechanically dissociated, and were subjected to enzymatic digestion by 20 mg/ml collagenase (Sigma-Aldrich) under a constant shacking at 37 °C for 20 min. The resultant cell suspension was then filtered using a 100 μm Nylon mesh (Thermo Fisher Scientific, Shanghai, China). The collected LCs were subjected to a series of purification steps involving 0–100% v/v Percoll linear density gradient (Sigma-Aldrich). The purity of LCs was verified using 3β-hydroxysteroid dehydrogenase (HSD3B) immunostaining (Riccetti, et al., 2017), along with qPCR analysis using primer sets specific to germ cells, Sertoli cells, and LCs marker genes (Sun, et al., 2013). The TM3 mouse Leydig tumor cell line, obtained from American Type Culture Collection (ATCC, Manassas, VA, USA), was routinely maintained in a 1:1 mixture of Ham's F12 medium and Dulbecco's modified eagle's medium supplemented with 2.5 mM L-Glutamine, 0.5 mM Sodium Pyruvate, 1.2 g/L sodium bicarbonate and 15 mM HEPES, 92.5% horse serum, 5% FBS (Thermo Fisher Scientific), as instructed by the manufacturer. The Stac3-null TM3 cells were generated by transfection of pLKO.1-CMV-tGFP-Stac3, followed by selection with 0.5 μg/ml G418 (Thermo Fisher Scientific). To study the hormone-stimulated steroidogenesis, LCs or TM3 cells were incubated for 12 h with 100 ng/ml luteinizing hormone (LH)/human chorionic gonadotropin (hCG), 0.5 mmol/l db-cAMP, 5 μmol/l 22R-hydroxycholesterol (22-ROH), or 5 μmol/l pregnenolone respectively (all reagents were obtained from Sigma-Aldrich), followed by hormone assay.

### Hormone assay

Serum testosterone levels in mice and concentrations of testosterone in the cell culture media were measured using a testosterone enzyme-linked immunosorbent assay (ELISA) kit (DRG, Marburg, Germany), as per the manufacturer's instructions. Final absorbance values were determined in triplicate at 450 nm on a microplate reader (Bio-Rad, Shanghai, China). The sensitivity of testosterone assay is 0.083 ng/mL. The intra-assay precision of these results is between 2.1 and 3.6%, and the inter-assay precision of is between 4.1 and 5.4%.

### RT-qPCR

Total RNA was extracted using E.Z.N.A.® HP Total RNA Kit (Omega Bio-tek, Guangzhou, China). Following routine DNase digestion for RNA purification, 800 ng of RNA was subjected to the synthesis of the first strand cDNA using the iScript™ cDNA Synthesis Kit (Bio-Rad). Subsequent qPCR analysis using ~ 10 ng cDNA/reaction was carried out according to the Promega protocol. The relative expression levels of target genes were calculated using the 2^−∆∆Ct^ method (Dong, et al., 2016), with amplification of *18S* as the internal control. The primers used were: mouse *Stac3* (NM_177707.4), 5′-CCCACTCTACAGCGACCAAC-3′ and 5′-GACTCTGGCTCCTCCTCCAT-3′; rat *stac3* (NM_001130558.1), 5′-ACTTCAGCTCTACCACCACG-3′ and 5′-CTCCATCTCGGTTGTCTCTGG-3′; human *STAC3* (NM_145064.3), 5′-TTTCAGCCCTGACACCAAGG-3′ and 5′-ACTTTGCCGAGTCTCTGCTG-3′; human *18S* (M10098.1, also applied to mouse and rat *18 s*), 5′-AATCAGGGTTCGATTCCGGA-3′ and 5′-GTGGACTCATTCCAATTACAG-3′. Primers used for *Pem*, *Star*, *Hsd3b* and *Cyp11a1* have been reported elsewhere (Wang, et al., 2006, 2018).

### Immunoblotting/Western blotting

Protein samples were prepared using a Chemicon® kit (Merck, Hong Kong, China). ~ 30 μg of protein samples were separated by SDS/PAGE, and were transferred to a PVDF membrane (Thermo Fisher Scientific). Membranes were then incubated at 4 °C overnight with different primary antibodies (Table 1). Positive signals were finally developed using the Immun-Star HRP chemiluminescence detection kit (Bio-Rad). Immunoblots hybridized with a preabsorbed serum (Abcam, Shanghai, China) served as negative controls.

**Table 1:** Antibodies used in this study

Antibodies	Property	Dilution	Source	Catalog number
STAC3	Rabbit Polyclonal	IB: 1:1000 IHC: 1:100 IF: 1:200	Thermo Fisher Scientific	PA5-75,753
TUBULIN	Rabbit Polyclonal	IB: 1:1000	Abcam	ab6046
StAR	Rabbit Polyclonal	IB: 1:1000	Novus	NBP1-33,485
Goat anti-Rabbit IgG (HRP)-second Ab	Goat	IB: 1:5000	Thermo Fisher Scientific	65–6120
Goat anti-Rabbit IgG (FITC)-second Ab	Goat	IB: 1:2000	Thermo Fisher Scientific	65–6111
VECTASTAIN® Elite® ABC HRP Kit	Peroxidase	IHC: 1:500	Vector Lab	PK-7200

*IB* immunoblotting/Western blotting, *IHC* immunohistochemistry, *IF* immunofluorescence

### Histological examination and measurement of testicular apoptosis

5-μm thick testicular sections were deparaffinized, rehydrated, and stained with hematoxylin–eosin for histological examination. To reveal the localization of STAC3 protein in situ, 5-μm thick testicular sections were deparaffinized, rehydrated, and subjected to antigen retrieval (20 min at 95 °C in 10 nM citrate buffer, pH 6.0). The sections were immersed in 0.5% v/v H_2_O_2_ /methanol for 20 min to block endogenous peroxidase activity. After PBS washes, sections were incubated at 4 °C overnight with STAC3 antibody. Subsequent hybridization with second-antibody and avidin–biotin complex (ABC) was performed with the aid of the VECTASTAIN ELITE® ABC Kit (Vector Lab, Burlingame, CA, USA). Peroxidases were finally detected with 0.7 mg/ml 3–3′-diaminobenzidine tetrahydrochloride (Sigma-Aldrich) in 1.6 mg/ml urea hydrogen peroxide.

Double-immunofluorescent cytochemistry was performed as described (Li, et al., 2014). TM3 cells were labeled with 500 nM MitoTracker Red CMXRos (Thermo Fisher Scientific) in fresh culture media at 37 °C for 30 min, and were then fixed with 4% paraformaldehyde for 15 min, followed by incubation at room temperature for 30 min with blocking solution (10% donkey serum, 0.5% bovine serum albumin and 0.1% triton X-100 in PBS). Cells were then incubated at 4 °C overnight with different antibodies (Table 1), followed by incubation at room temperature with fluorescein isothiocyanate (FITC)-labeled and rhodamine-labeled second antibodies. Cells were finally incubated at room temperature with 4,6-diamidino-2-phenylindole (DAPI; Sigma-Aldrich) for 10 min. Double-immunofluorescent signals were observed and recorded under an Axio Imager M1 inverted microscope (Zeiss, Shanghai, China).

Testicular apoptosis was assessed using an apoptosis enzyme-linked immunosorbent assay (ELISA) kit (Roche, Shanghai, China), following the manufacturer’s instructions.

### Analysis of ΔΨ_m_ with TMRE

Mitochondrial ΔΨ_m_ in stimulated LCs was evaluated by measuring uptake and accumulation of the potentiometric tetramethylrhodamine ethyl ester dye (TMRE, Sigma-Aldrich) (Allen, et al., 2006). TM3 cells were cultured to a maximum of 75% confluency in a 96-well florescence assay plate (Thermo Fisher Scientific). After incubation with 0.5 mM db-cAMP for 6 h, cells were treated at 37 °C in 200 μl of a 50-nM solution of TMRE in serum-free medium for 20 min. Final TMRE fluorescence was measured using an excitation of 550 nm (540/25 nm filter) and an emission at 590 nm (590/20 nm filter) under the Axio Imager M1 inverted microscope (Zeiss).

### Northern blot

A biotin-labeled RNA probe was produced by in vitro transcription reactions using Ambion's MAXIscript™ Kit, following the manufacturer’s instructions. RNA samples were prepared as described above. About 30 μg of RNA samples were separated by denaturing agarose gel electrophoresis, and were then transferred onto a Blotting-Nylon 66 membrane (Sigma-Aldrich). Subsequent Northern blot analysis was carried out with the aid of the NorthernMax™ and BrightStar™ BioDetect™ Nonisotopic Detection system (Thermo Fisher Scientific). Final visualization of positive signals was achieved through phosphorimaging (Typhoon, Mundelein, IL, USA).

### Statistical analysis

Data are presented as mean ± S.E.M. Statistical significance was assessed by performing *Student's t*-test or one way analysis of variance (ANOVA) as appropriate, using a *P-*value of 0.05. Statistical analyses were performed with SPSS13.0 software package (SPSS Inc., Chicago, IL, USA).

## Results

### Testicular STAC3 is exclusively expressed by LCs

**Fig. 1:**
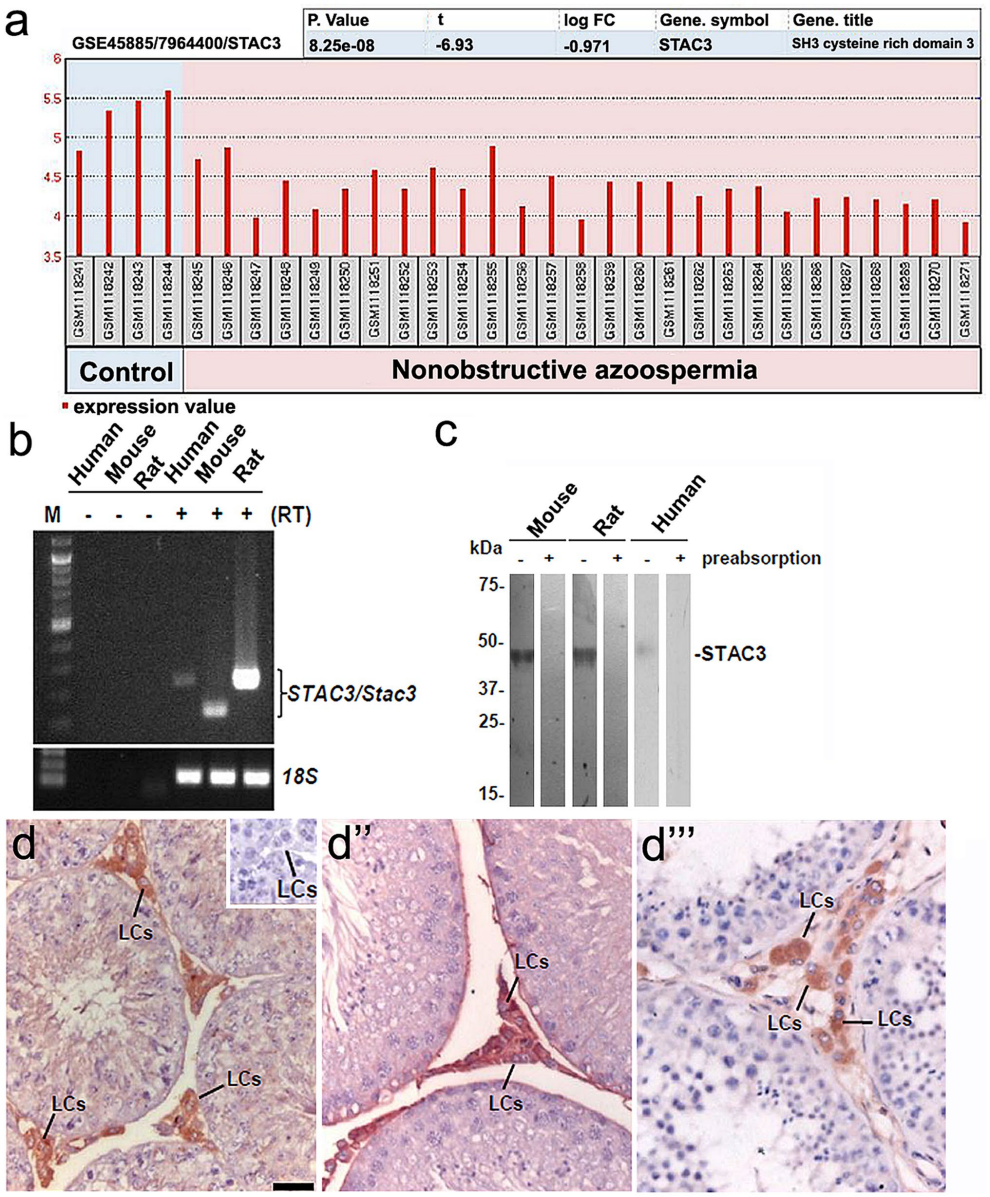
Expression of STAC3 in rodent and human testes. **(a)** Curve of analytical results showing the expression levels of *STAC3* transcripts in testicular biopsies from 4 normozoospermia and 27 nonobstructive azoospermia, as reported by GEO Database (GSE45885). **(b)** Representative RT-PCR assay of the expression levels of *Stac3*/*STAC3* mRNA in rodent and human testes was presented. Amplification of *18S* mRNA served as the internal control. The PCR assay without reverse transcription was also accompanied as negative control. **(c)** Immunoblotting analysis demonstrated a single band of STAC3 protein in the testicular lysates from adult mouse, rat and human, whereas preabsorption with blocking peptides totally abolished the positive immunostaining. **(d-d’’)** Sections of adult rodent and human testes immunostained with a rabbit anti-STAC3 polyclonal antibody and counterstained with hematoxylin. Clear immunostaining is observed in the testicular interstitium. d, mouse testis; d’, rat testis; d’’, human testis. LCs, Leydig cells. Inserted panel, immunostaining with blocking peptides totally abolished the positive staining. Bar = 50 μm

From the transcriptomic profiles of 4 normozoospermia and 27 non-obstructive azoospermia deposited in the GEO database (GSE45885), the expression levels of the *STAC3* transcripts, were observed to be significantly downregulated in non-obstructive azoospermia compared to those in normozoospermia (*P* < 0.0001, Fig. 1a). This finding suggests that the expression of *STAC3* may be associated with spermatogenesis. As an initial step to understand the function of STAC3, we established the expression pattern of STAC3 in testis. RT-PCR assay revealed a distinct expression of *Stac3*/*STAC3* in rodent and human testes (Fig. 1b). Moreover, immunoblotting analysis demonstrated a single band of STAC3 protein in the whole blot, whereas blots treated with a preabsorbed primary antibody demonstrated no bands, confirming the assay specificity (Fig. 1c). Subsequent immunohistochemistry showed that testicular STAC3 is exclusively expressed by LCs (Fig. 1d, d’ and d’’).

### Verification of the STAC3 localization in rodent LCs

**Fig. 2:**
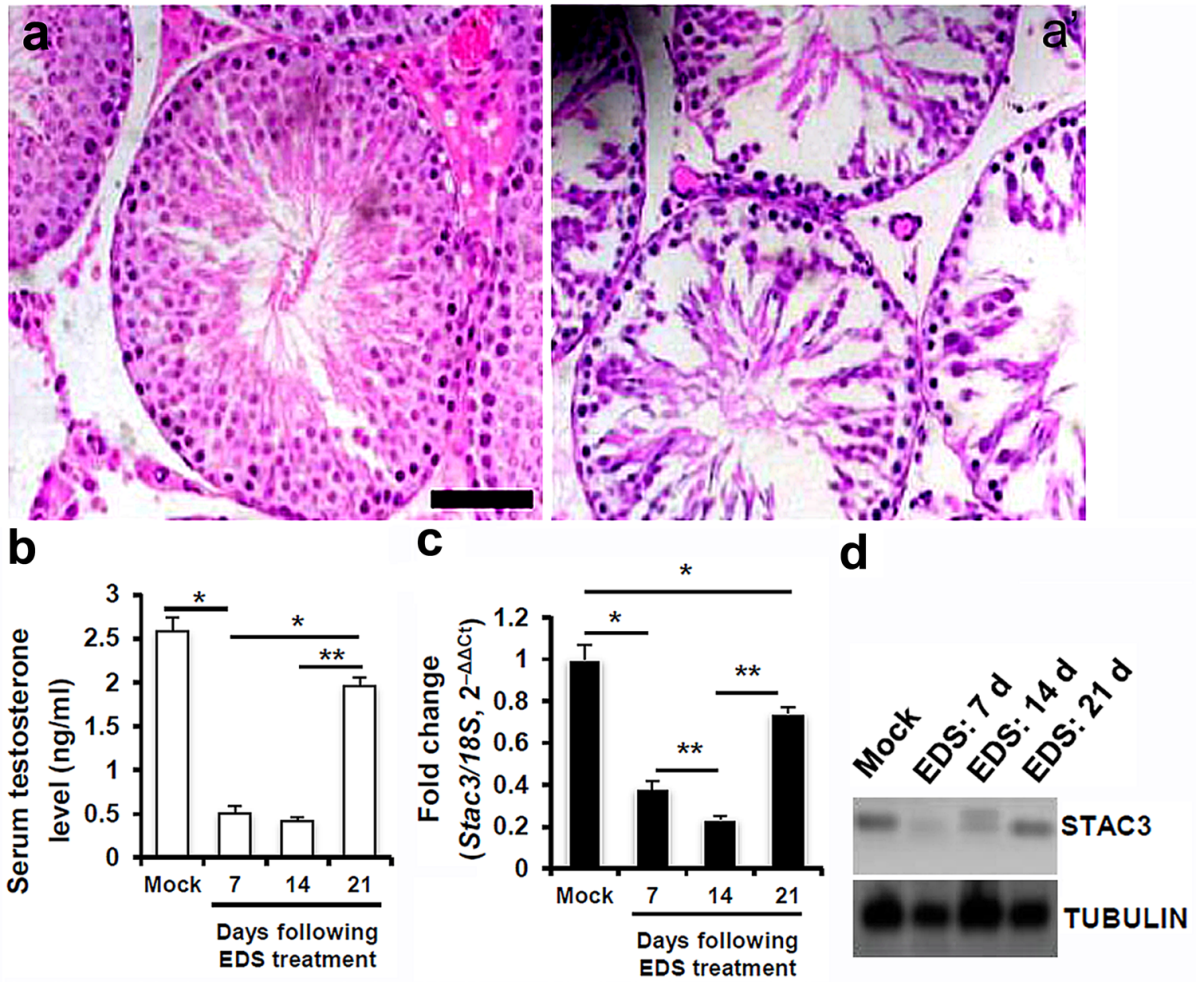
Verification of the STAC3 localization in LCs using a cytotoxic drug ethylene dimethane sulfonate (EDS). **(a-a’)** Representative H&E-stained transverse testis sections showing morphology at a, 0; a', 7 d after administration of EDS. Bar = 100 μm **(b)** Serum testosterone level (ng/ml) in EDS-treated rats was evaluated as described in Materials and Methods (*P < 0.05 and **P < 0.01). **(c)** The expression levels of *Stac3* mRNA in rat testis at different time points following EDS treatment were assessed by RT-qPCR. Relative expression levels were determined in which the level of a target mRNA was normalized against the mRNA level in mock control, which was arbitrarily set at 1. Data were presented as the mean ± S.E.M. of at least 3 determinations. **(d)** Levels of STAC3 protein in rat testis at different time points following EDS treatment were assessed by immunoblotting. TUBULIN was used as the loading control

Because STAC3 expression in mammalian testes appeared to be relatively conserved, we used EDS-treated rat testis to further verify the localization of STAC3 in LCs. Elimination of LCs by EDS was confirmed by histological examination (Fig. 2a and a’) and by measurement of circulating testosterone concentrations (Fig. 2b). In accordance with the previous reports (He, et al., 2016; Tena-Sempere, et al., 2002), testosterone concentrations in EDS-treated rats dropped to nearly undetectable levels at d 7 and 14, and began to restore at d 21 following EDS administration. Intriguingly, testicular expression of STAC3 was significantly decreased at d 7 and 14 after EDS injection, with the lowest values being observed at d 14. By contrast, expression of STAC3 restored substantially at 21 following EDS injection, well consistent with the hormonal changes (Fig. 2c and d). Thus, testicular expression of STAC3 is exclusively enriched in LCs.

### Effects of in vivo inhibition of STAC3 expression on testicular steroidogenesis and spermatogenesis

**Fig. 3:**
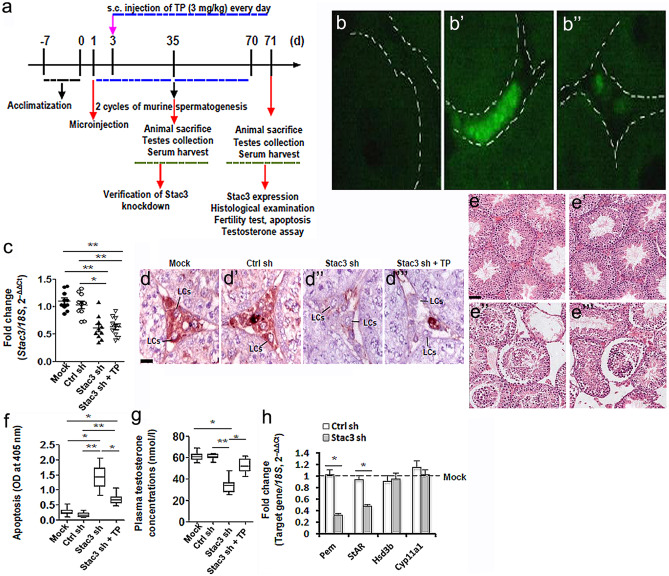
Effects of in vivo inhibition of STAC3 on testicular morphology, germ cell apoptosis, androgen production and male fertility. **(a)** Schematic representation of the experimental procedures used in the in vivo lentiviral vector–mediated shRNA treatment. **(b-b’’)** Enrichment of lentiviral shRNA in the testicular interstitium following microinjection was demonstrated by observing frozen sections under a fluorescence microscope. b, mock; b’, 35 d after microinjection; b’’, 70 d after microinjection. **(c)** Inhibition of *Stac3* in testis by in vivo shRNA treatment was confirmed by RT-qPCR at 70 d after microinjection. Data were presented as the mean ± S.E.M. of at least 3 determinations (n = 10, *P < 0.05 and **P < 0.01). **(d-d’’’)** Immunostaining of STAC3 protein in testicular sections was carried out at different time points following microinjection. Bar = 25 μm **(e-e’’’)** Representative H&E-stained transverse testis sections showing morphological changes at different time points following microinjection. e, mock; e’, Ctrl sh; e’’, Stac3 sh; e’’’, Stac3 sh + testosterone propionate (TP). Bar = 50 μm **(f)** Effects of in vivo shRNA treatment on germ cell apoptosis at 70 d after microinjection were assessed using an ELISA kit (n = 10, *P < 0.05 and **P < 0.01). **(g)** Plasma testosterone levels (nmol/l) in mice from different groups at 70 d after microinjection were assessed using an ELISA method (n = 10, *P < 0.05 and **P < 0.01). (**h**) Effects of inhibition of *Stac3* in testis by in vivo shRNA treatment on different target transcripts were revealed using RT-qPCR at 70 d after microinjection. Data were presented as the mean ± S.E.M. of at least 3 determinations (n = 5, *P < 0.05)

It is becoming increasingly evident that microinjection of lentiviral plasmids could achieve an efficient in vivo gene transfer in LCs (either ectopic overexpression or targeted knockdown). This newly developed method therefore is a valuable tool to address the function of specific genes in testicular steroidogenesis (Kim, et al., 2010; Park, et al., 2013). To study the potential role of STAC3 in LCs at the in vivo level, we microinjected pLKO.1-CMV-tGFP-Stac3 or empty vector into the seminiferous tubules of adult C57BL/6 J mice. We then studied the expression of Stac3, testicular histology, fertility potential, testicular apoptosis and plasma testosterone concentrations in different experimental groups (Fig. 3a). Using a GFP reporter allele to evaluate targeting efficiency and specificity, we observed that the lentivirus-mediated bioluminescence was strongly enriched in the testicular interstitium at 35 d and was moderately expressed in the testicular interstitium at 70 d following microinjection, compared to the negative expression of bioluminescence in untreated testis (Fig. 3b, b’ and b’’). In accordance with these findings, at 70 d following microinjection, *Stac3*/STAC3 expression was significantly reduced in the testes injected with pLKO.1-CMV-tGFP-Stac3 compared with mock testes or those injected with empty vector, as revealed by RT-qPCR (Fig. 3c) and immunohistochemistry (Fig. 3d, d’, d’’ and d’’’). By contrast, supplement with testosterone propionate by s.c. injection on a daily basis failed to rescued the *Stac3*/STAC3 expression levels in the lentivirus-treated testes, suggesting that testicular expression of STAC3 is not regulated by androgen signaling. At 70 d following microinjection, mock testes exhibited normal histological features and high Johnsen’s scores, while Stac3 shRNA-treated testes demonstrated degenerative changes including germ cells desquamation, decrease of germinal epithelium height, and lower Johnsen’s scores (Fig. 3e, e’, e’’ and e’’’, Table 2). Consequently, inhibition of STAC3 expression caused impaired male fertility by inducing oligozoospermia and asthenospermia (Table 2). The stimulatory effect of *Stac3* shRNA on testicular apoptosis, along with its inhibitory effect on androgen production, was also detected by ELISA at 70 d following microinjection (Fig. 3f and g). More importantly, the deleterious effects of *Stac3* shRNA on testicular histoarchitecture, epididymal parameters, fertility potential, and testosterone production, were all successfully ameliorated by co-treatment with testosterone propionate (Fig. 3e, f, g and Table 2). For the selected genes expressed in testis, we observed that the expression levels of *Pem* (a well-defined androgen target gene) (Wang, et al., 2006) and *Star* (the first rate-limiting enzyme in testosterone synthesis) (Wang, et al., 2018) were reduced upon STAC3 ablation, whereas transcriptional levels of other rate-limiting enzymes (namely *Hsd3b* and *Cyp11a1*) were comparable in Ctrl sh and *Stac3* sh-treated testes (Fig. 3h). These findings collectively suggest that STAC3 may regulate spermatogenesis by targeting the testicular steroidogenesis.

**Table 2:** Histomorphometry, epididymal parameters and assessment of male fertility 70 d after the first microinjection with *Stac3* shRNA lentivirus

Parameters		Experimental groups			
		Mock	Ctrl sh	Stac3 sh	Stac3 sh + TP
Johnsen’s score		9.17 ± 0.18^a^	9.06 ± 0.24^a^	2.87 ± 0.63^b^	5.42 ± 0.46^c^
Germinal epithelium height (μm)		57.42 ± 1.79^a^	58.11 ± 2.31^a^	21.05 ± 1.98^b^	37.65 ± 2.64^c^
Cauda sperm concentration (Million)		45.94 ± 2.37^a^	47.04 ± 1.98^a^	26.28 ± 2.73^b^	35.24 ± 3.13^c^
Sperm motility (%)		65.14 ± 2.06^a^	63.84 ± 3.12^a^	25.74 ± 1.33^b^	59.67 ± 2.29^a^
Fertility	Pregnancies/females mated	42/46(91.3%)^a^	38/44(86.4%)^a^	11/34(32.4%)^b^	21/39(53.8%)^c^
	Litter size	8.8 ± 0.9^a^	9.0 ± 0.7^a^	3.2 ± 1.1^b^	5.4 ± 0.6^c^

Data were analyzed by one-way ANOVA followed by Tukey's test for multiple comparisons and were expressed as mean ± SEM. *TP* testosterone propionate. Different alphabet letter above the bar graph indicates statistically significant differences between groups (a, b, c, d: *P* < 0.05)

### Validation of indispensible role of STAC3 in testicular steroidogenesis using the in vivo knockdown model with isolated primary LCs as well as in vitro experiments with TM3 and primary LCs

**Fig. 4:**
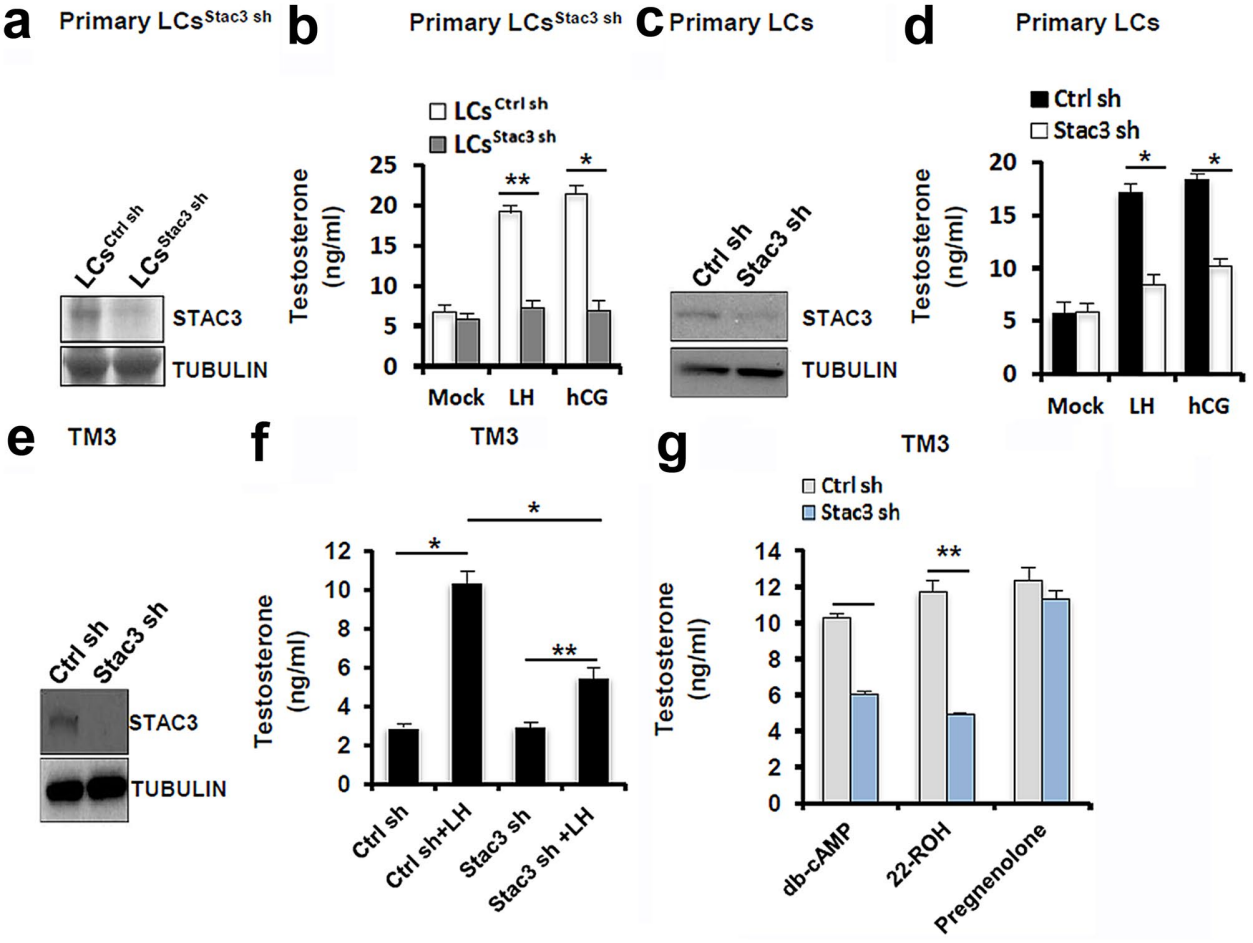
Effects of inhibition of STAC3 on basal and LH-stimulated testosterone production in primary LCs and TM3 cells. **(a)** Primary LCs were isolated and purified from Ctrl sh or *Stac3* sh-treated testes at 70 d after microinjection. The inhibition of STAC3 in testis was confirmed using immunoblotting. **(b)** Primary LCs^Stac3 sh^ or Ctrl LCs were treated with 100 ng/ml luteinizing hormone (LH)/human chorionic gonadotropin (hCG) for 12 h, followed by measurement of testosterone concentrations in the culture media using an ELISA method (n = 4, *P < 0.05 and **P < 0.01). **(c)** Primary LCs isolated from wild-type testis were transfected with *Stac3* shRNA or Ctrl shRNA. 48 h later, LCs were treated with 100 ng/ml LH/hCG for 12 h, followed by measurement of testosterone concentrations in the culture media using an ELISA method (n = 4, *P < 0.05 and **P < 0.01). **(e)** Generation of the TM3^Stac3−/−^ cells was confirmed by immunoblotting. **(f)** TM3^Stac3−/−^ or control cells were treated with 100 ng/ml luteinizing hormone (LH) for 12 h, followed by measurement of testosterone concentrations in the culture medium using an ELISA method (n = 4, *P < 0.05 and **P < 0.01). **(g)** Testosterone production stimulated for 12 h by db-cAMP (0.5 mM), 22R-hydroxycholesterol (22-ROH, 5 μM), or pregnenolone (5 μM) in the TM3^Stac3−/−^ or control cells was determined as described. Quantitative values are means ± S.E.M. (n = 4, *P < 0.05 and **P < 0.01)

To validate the in vivo data, we isolated the primary LCs from Ctrl shRNA or *Stac3* sh-treated testes (Supplementary Fig. 1, Fig. 4a). LCs were treated with LH and hCG for 12 h and testosterone concentrations in the culture media were then measured. STAC3 deficiency significantly impaired the gonadotropin-induced testosterone synthesis (Fig. 4b). To determine whether transient knockdown of STAC3 also affected testicular steroidogenesis, we tranfected the primary LCs from WT testes with Ctrl shRNA or *Stac3* shRNA (Fig. 4c). Subsequent hormone assays revealed that transient knockdown of STAC3 also attenuated the gonadotropin-induced testosterone synthesis in LCs (Fig. 4d). Thus, endogenous expression of STAC3 is required for steroidogenesis in LCs. To further study the functional details of STAC3, we generated the TM3^Stac3−/−^ cells (Fig. 4e). The resultant TM3^Stac3−/−^ cells were stimulated for 12 h with 100 ng/ml LH. Apparently, STAC3 depletion caused a ~ 46.7% decrease in the testosterone secretion in the LH-stimulated cells, but had no effects on the basal testosterone secretion in vitro (Fig. 4f). To identify at which step the steroidogenic process was altered by STAC3 deficiency, TM3^Stac3−/−^ cells were stimulated with different stimulus including db-cAMP, 22-ROH or pregnenolone. ELISA assays using cell culture supernatants showed that testosterone secretion was significantly reduced in db-cAMP- or 22-ROH-treated TM3^Stac3−/−^ cells, but remained unaffected in pregnenolone-treated TM3^Stac3−/−^ cells (Fig. 4g). STAC3 depletion thus may sabotage steroidogenesis by inducing mitochondrial dysfunction.

### STAC3 depletion impairs mitochondrial membrane potential and StAR processing in db-cAMP-stimulated LCs

**Fig. 5:**
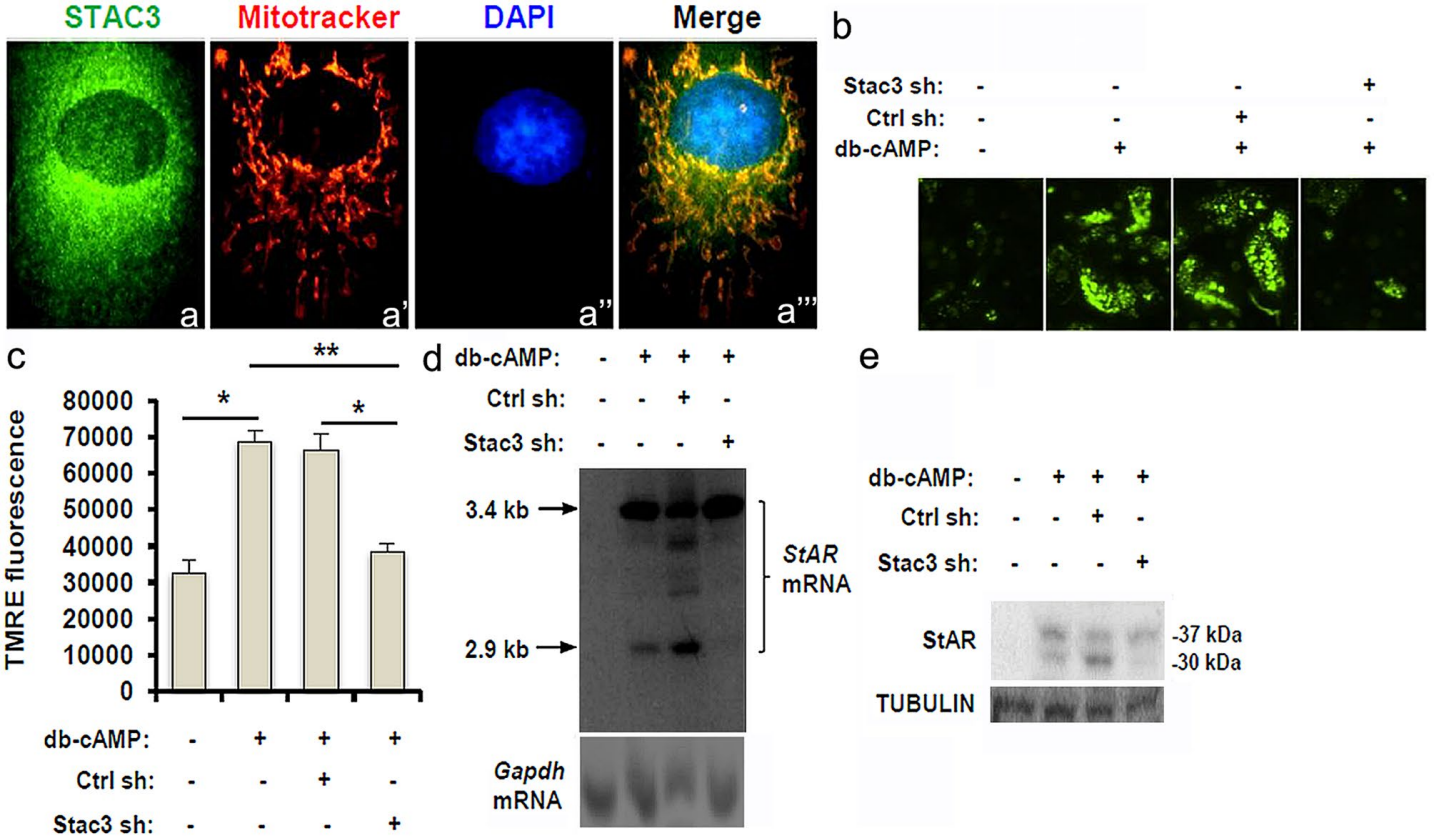
Effects of inhibition of STAC3 on mitochondrial membrane potential and StAR processing in TM3 cells. **(a-a’’’)** TM3 cells were labeled with Rhodamine Mitotracker, followed by immunofluorescence staining with an anti-STAC3 antibody. Nuclei were counterstained using DAPI. Localization of STAC3 was determined by confocal microscopy. Yellow color denotes the colocalization of STAC3 and Mitotracker. **(b)** TM3 cells were cultured to ~ 75% confluency in a 96-well florescence assay plate. After incubation with 0.5 mM db-cAMP for 6 h, cells were incubated in 200 μl of a 50-nM solution of TMRE in serum-free medium for 20 min at 37 °C. Uptake of TMRE was then determined by observing TMRE fluorescence under a fluorescence microscope. **(c)** Quantification of TMRE fluorescence in Penal (b) using a florescence assay plate reader. Data are represented as mean ± S.E.M. from 5 independent experiments. **(d)** The TM3 cells with different transfections were treated with 0.5 mM db-cAMP for 6 h, followed by non-radioactive Northern blot analysis to measure the changes in the expression of *StAR* mRNA. **(e)** TM3 cells with different transfections were treated with 0.5 mM db-cAMP for 6 h, followed by immunoblotting analysis to assess the changes in the expression of StAR protein. TUBULIN was used as the loading control

Having establishing the functional link between STAC3 expression and mitochondrial dysfunction, we tried to identify the potential molecular basis underpinning our observations. When we investigated the cellular localization of STAC3 in TM3 cells using double-immunofluorescent cytochemistry, STAC3 was observed to be perfectly overlapped with Mitotracker labeling in the cytoplasm (Fig. 5a, a’a’’ and a’’’). A polarized mitochondrial potential is absolutely necessary for steroidogenic activation in LCs, and the magnitude of *ΔΨ*_*m*_ can be determined by measuring the uptake of TMRE dye in mitochondria (Allen, et al., 2006). We treated the TM3 cells with 0.5 M of db-cAMP to acutely stimulate steroidogenesis and then subjected these cells to TMRE uptake assay. Treatment with db-cAMP induced a significant increase in the uptake of TMRE fluorescence in naïve or Ctrl shRNA-treated TM3 cells, but failed to stimulate noticeable steroidogenic activity in the TM3^Stac3−/−^ cells (Fig. 5b and c). StAR is a rapidly synthesized transport protein in response to intracellular pulses of cAMP. StAR protein regulates the rate-limiting step in steroidogenesis, *i.e.* the delivery of cholesterol from the outer (OMM) to the inner (IMM) mitochondrial membrane. (Hiroi, et al., 2004). To ask whether the inhibitory effects of STAC3 deficiency on steroidogenesis occurred at the level of StAR, we measured the changes in *StAR* mRNA and StAR protein. Treatment with db-cAMP induced the expression of the 3.4-kb precursor form of *StAR* mRNA as well as the 2.9-kb processed form in naïve or Ctrl shRNA-treated TM3 cells. Consistently, db-cAMP stimulated the expression of the 37-kDa precursor form of StAR protein as well as the 30-kDa processed form in both cells. By contrast, STAC3 depletion significantly decreased the processed forms of *StAR* mRNA (2.9-kb) and protein (30-kDa) upon db-cAMP challenge (Fig. 5d and e). These data together suggest that endogenous STAC3 in LCs is required for mitochondrial membrane potential and StAR processing in the acute regulation of testosterone biosynthesis.

## Discussion

Testicular steroidogenesis is under a delicate control of a cohort of signals from mitochondria. The changes in the intratesticular environment have been observed to influence the mitochondrial homeostasis and dynamics in testicular steroidogenesis (Bjelic, et al., 2015; Radovic, et al., 2019; Shen, et al., 2016; Strauss, et al., 2009). Nevertheless, the key factors responsible for the modulation of mitochondrial homeostasis in activated LCs remain largely unexplored. In the current study, we tested the hypothesis that STAC3, an adaptor protein that is essential for excitation–contraction coupling and calcium channel function in skeletal muscle (Reinholt, et al., 2013), might be functionally expressed in testis. We have shown that STAC3 was expressed with high selectivity in rodent and human LCs (Fig. 1). In agreement, STAC3 expression was negligible in rat testis after selective withdrawal of LCs by administration of the cytotoxic drug EDS, and reappeared immediately after repopulation of LCs (Fig. 2). Thus, LCs serve as the main source of testicular expression of STAC3.

A previous study using microarray analysis has identified STAC3 as one of the most significantly down-regulated genes in infertile male patients. These findings point to a correlation between deregulated expression of STAC3 and spermatogenic failure (Malcher, et al., 2013). Apparently, we could not rule out the possibility that the pathological changes within seminiferous tubules from infertility patients may be detrimental to STAC3 expression. To address this, we employed an in vivo lentiviral vector–mediated microinjection which has been validated to be successful to achieve ectopic gene transfer in LCs (Kim, et al., 2010; Park, et al., 2013). Our results reveal that inhibition of STAC3 expression significantly impaired male fertility by inducing oligozoospermia and asthenospermia, and this impairment can be ascribed to androgen deficiency (Fig. 3, Table 2). Because the immortalized TM3 cells do not fully recapitulate the behavior of endogenous LCs, we validated the indispensible involvement of STAC3 in testicular steroidogenesis using the in vivo knockdown model with isolated primary LCs as well as in vitro experiments with primary cultured LCs (Fig. 4). It is gradually recognized that motion system and male reproduction are coordinately regulated. For example, osteoblast-derived hormone osteocalcin promotes testosterone production and germ cell survival in a CREB-dependent manner (Oury, et al., 2011). By contrast, body wall muscle-produced SWM-1 negatively regulates sperm activation in *C. elegans* by targeting TRY-5, a spermiogenesis activator (Chavez, et al., 2018). These studies suggest that both bone and skeleton muscle are endocrine organs, and their remodeling throughout adulthood may be tightly associated with male reproductive processes. Our findings extend these understandings by identifying STAC3 as a novel modulator of testicular steroidogenesis. Of note, the mechanisms whereby STAC3 expression is sabotaged in testis with spermatogenesis arrest remain to be defined, one factor has been so far reported to negatively regulate the expression of STAC3. The cyclic adenosine monophosphate (cAMP) is a well-known second messenger that is important in mediating both basal and hCG-stimulated testosterone release in LCs (Kong, et al., 2017). However, abnormal accumulation of cAMP could significantly inhibit testosterone production (Tsai, et al., 1996). Thus, a proper balance in the production of cAMP is likely involved in normal steroidogenesis. Interestingly, there is increasing evidence that accumulation of cAMP correlates to decreased calcium channel activity during impaired testicular steroidogenesis (Tsai, et al., 1997). In light of the critical role of STAC3 in the regulation of calcium channel function (Campiglio, et al., 2018; Campiglio and Flucher, 2017), it will be of future interest to deconvolute if a cAMP/STAC3/Ca^2+^ axis is also at play in LCs.

By generating the TM3^Stac3−/−^ cells, we further provided the molecular evidence that STAC3 depletion attenuated mitochondrial membrane potential and StAR processing in the db-cAMP-stimulated LCs (Fig. 5). STAC3 was originally identified as a critical EC coupling regulator that triggers contraction of the sarcomere by converting electrical signals to Ca^2+^ transients (Nelson, et al., 2013). So it is a logical observation that endogenous STAC3 is indispensible for an intact mitochondrial potential. Moreover, ablation of STAC3 expression decreased the processed form of StAR expression at both mRNA and protein levels (Fig. 5d and e). This observation is intriguing, as compared to the previous findings that that disrupting mitochondria usually leads to posttranscriptional changes in StAR during testicular steroidogenesis (Allen, et al., 2006). It is therefore possible that STAC3 may incorporate a transcriptional network to modulate the dynamics of StAR expression in LCs. In favor of our hypothesis, STAC3 has been shown to inhibit the proliferation of pulmonary artery smooth muscle cells (PASMCs) by promoting the deacetylation of FOXO1 transcription factor or by potentiating the activation of PGC-1α, a master coactivator of mitochondrial biogenesis (Zurlo, et al., 2018). The physiological relevance of the crosstalk between STAC3 and its interacting transcription factors in stimulated LCs is presently under evaluation in our lab.

**Fig. 6:**
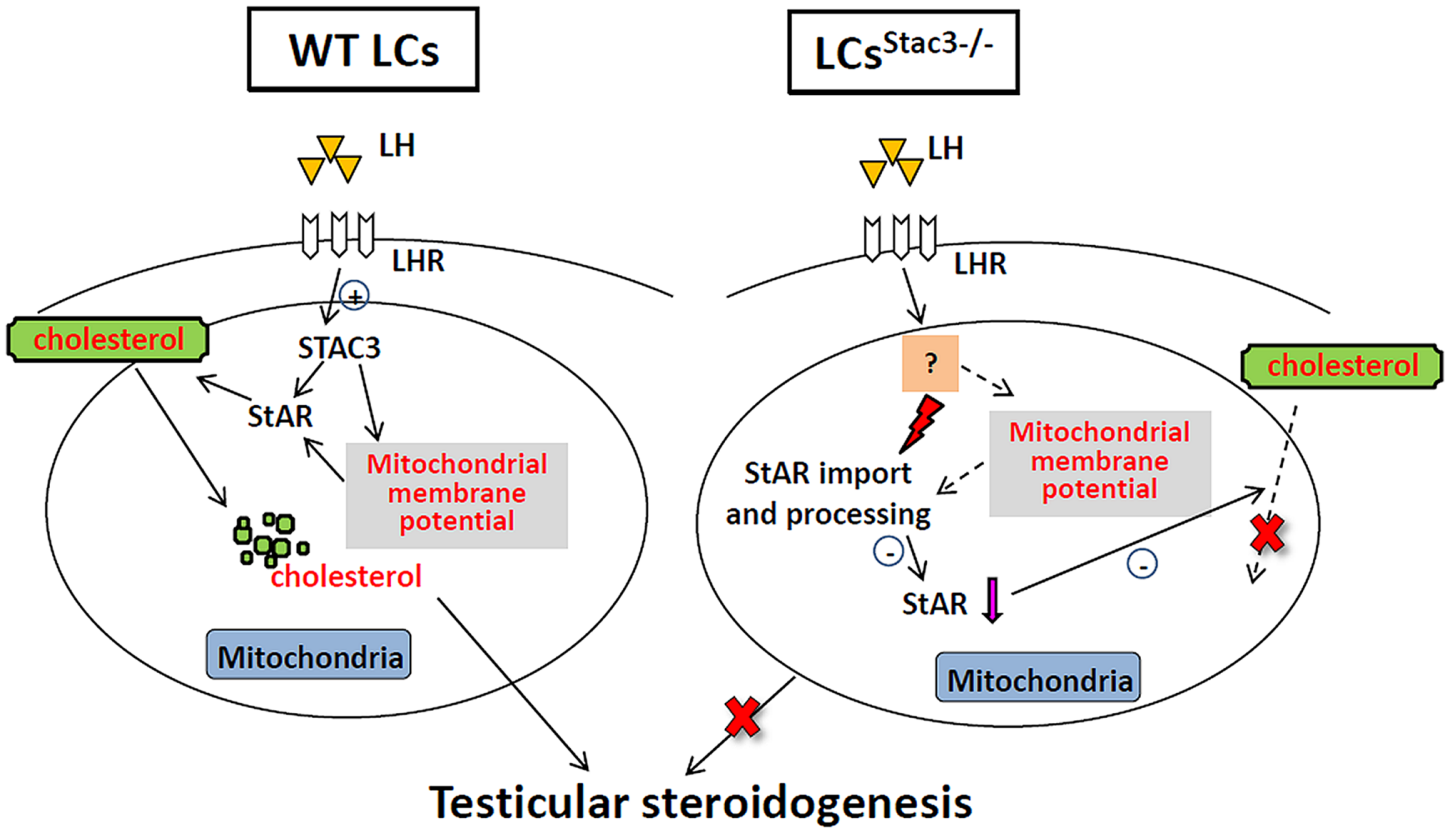
Proposed model of the STAC3 signaling pathway in the regulation of mitochondrial membrane potential, StAR processing and testosterone synthesis in LCs

In conclusion, our data demonstrate that the EC coupling regulator STAC3 is expressed in the mitochondria of testicular LCs. We provide the first evidence for a direct involvement of STAC3 in the control of testosterone production by maintaining mitochondrial membrane potential and StAR processing (Fig. 6). Overall, the available data strongly suggest that testicular STAC3 may function as a novel regulator linking mitochondrial homeostasis and testicular steroidogenesis.

## Electronic supplementary material

Below is the link to the electronic supplementary material.

Supplementary file1 (TIF 2.88 MB)
