# Supplementary figures and images for: Testicular STAC3 regulates Leydig cell steroidogenesis through potentiating mitochondrial membrane potential and StAR processing
Source: Cell Tissue Res. 2021 Jan 6;384(1):195–209. doi: 10.1007/s00441-020-03312-8 (PMC8016781; doi:10.1007/s00441-020-03312-8)

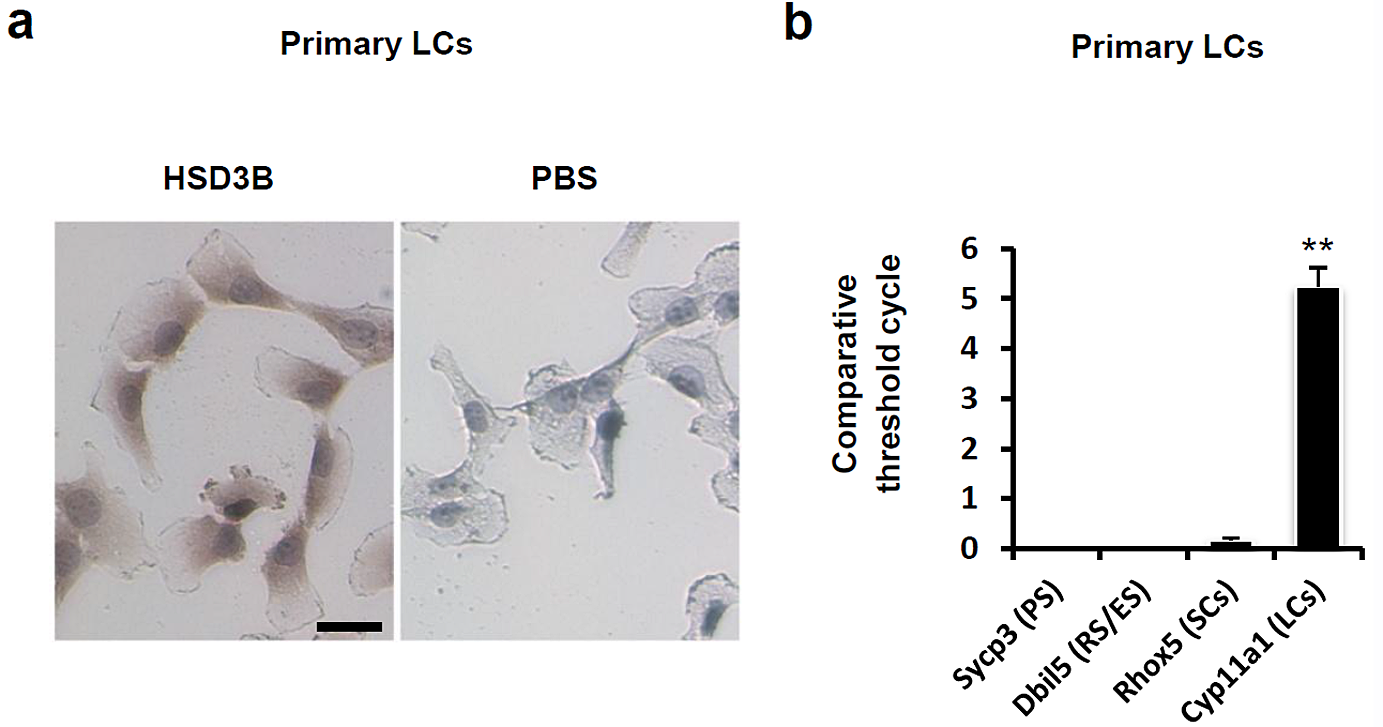

Supplement: Supplementary file 1 — Supplementary file1 (TIF 2.88 MB) [file 441_2020_3312_MOESM1_ESM.tif]
